# Morphological and skill-related fitness components as potential predictors of injury in elite netball players: A cohort study

**DOI:** 10.4102/sajp.v77i1.1524

**Published:** 2021-05-18

**Authors:** Colleen J. Sinclair, Frederik F. Coetzee, Robert Schall

**Affiliations:** 1 Department of Exercise and Sport Sciences, Faculty of Health Sciences, University of the Free State, Bloemfontein, South Africa; 2 Department of Mathematical Statistics and Actuarial Sciences, Faculty of Natural and Agricultural Sciences, University of the Free State, Bloemfontein, South Africa

**Keywords:** netball, risk factors, injury, prevalence, incidence, skill-related fitness

## Abstract

**Background:**

A limited number of studies on the epidemiology of injuries and fitness profiles of netball players in South Africa have been conducted, but no research on the potential morphological and skill-related fitness predictors of injuries could be located.

**Objectives:**

We investigated whether morphological or skill-related factors measured in the pre-season could predict injuries sustained in-season.

**Method:**

In our cohort study, 77 under-18 (U18), U19, U21 and senior elite netball players underwent pre-season testing including anthropometry, balance, flexibility, explosive power, upper and lower body strength, core strength, speed and agility testing. A questionnaire was used to collect demographic data, elite-level experience and injury history. Injuries in pre-season, training and matches were recorded during the subsequent 2017–2018 season using an injury profile sheet.

**Results:**

Amongst the 77 players who underwent pre-season fitness tests, 33 players (42.9%) had at least one injury. Regarding player morphology, a significant association of body mass and body fat percentage with injury risk was found in a simple logistic regression. In a multiple logistic regression analysis, only fat percentage (*p* = 0.0508) remained a significant predictor of injury at the 10% significance level, with higher fat percentage being associated with lower injury risk.

**Conclusion:**

Heavier players and players with a higher fat percentage had a decreased injury risk.

**Clinical implications:**

As a result of the apparent protective effect of heavier weight of players, referees should more strictly enforce the no-contact rule in netball. Further research on functional movement screening as a tool for potential prediction of injury in netball is recommended.

## Introduction

Netball is a popular sport among women, particularly in countries of the Commonwealth of Nations. According to the International Federation of Netball Associations (IFNA), over 20 million people play netball in more than 72 countries.

Netball is played at high intensity and requires speed, agility, power, dynamic balance and proprioception (Bourdon et al. 2017; Chandler et al. 2014; Coetzee, Langeveld & Holtzhausen 2014; McKenzie, Whatman & Brughelli 2019; Shaw et al. 2020). Furthermore, many games are played on cement surfaces, which puts greater physiological stress on the player, specifically the musculoskeletal system and the lower limb joints and ultimately increases the risk of injury (Langeveld, Coetzee & Holtzhausen 2012). These demands result in a high incidence of injury, mostly to the lower leg and particularly to the knee and ankle (Attenborough et al. 2017; Ellapen et al. 2015; Pillay & Frantz 2012; Reid et al. 2015; Thomas et al. 2017).

Soh et al. (2008) states that greater weight, height and fat percentage decrease the risk of injury. Similarly, a study conducted by Ferreira and Spamer (2010) shows that decreased explosive power and increased biomechanical deviations increase the risk of injuries. Attenborough et al. (2017) report an increased probability of sustaining an ankle sprain during netball when the distance reached in the posterior-medial direction of the star excursion balance test is less than or equal to 77.5% of leg length (Odds ratio [OR] = 4.04, 95% confidence interval [CI] = 1.00–16.35). McManus, Stevenson & Finch (2006) identified two risk factors and two protective factors that increase or reduce the risk of injury in netball players. The risk factors associated with increased risk of injury are not warming up before a game (incidence rate ratio [IRR] 1.11, 95% CI 1.00–1.23); training for at least 4 h per week had fewer injuries than those who did not (IRR 0.55, 95% CI 0.45–0.98) and players who did not sustain an injury in the preceding 12 months were at a lower risk of injury (IRR 0.58, 0.43–0.79) (McManus et al. 2006).

The centre (C), goal attack (GA) and wing defence (WD) have the highest risk of injury, whilst the goal-keeper (GK) has the smallest (Sinclair, Coetzee & Schall 2020b). Similarly, Smith et al. (2005) report that GA, wing attack (WA) and goal defence (GD) suffer the most injuries and GK the least. In contrast, McManus et al. (2006) report that defensive injuries (22.8% by either the GK or GD) are more likely to occur than any other injuries elsewhere on the court.

Furthermore, each sport has its specific physiological demands that coaches need to consider while preparing for matches. These physiological demands are determined by the rules and structure of the game, as well as the skill, tactical play and intrinsic ability of players (Thomas et al. 2016). Pillay and Frantz (2012) suggest that the most common knee and ankle injuries occur because of incorrect landing techniques, tripping and playing on outdoor cement surfaces. Furthermore, Ellapen et al. (2015) report poor landing (37.8%), being pushed by an opponent (22.7%), colliding with a team-mate (20.8%) and fast rotational movement (18.5%) as mechanisms of injury.

Players and coaches should have a thorough understanding of the game and be able to cope with the physical demands of the sport-specific requirements (Thomas et al. 2016). Various fitness tests can be conducted to determine a netball player’s physiological profile (Ahmuin 2013; Hotter, Crockett & Clancy 2014; McKenzie et al. 2019; Soh, Husain & Soh 2006). These tests identify the players’ strengths and weaknesses and provide the strength and conditioning coach with information regarding the effectiveness of their training programmes.

The high incidence of injury reported in netball requires preventative strategies based on the results of epidemiological studies. Preventative strategies and activities are necessary for both medical and financial reasons. Identifying variables measured during pre-season or pre-participation physical examinations and tests that might predict an increased risk of injury has been a goal of athletic programmes for the past 20–30 years (Sanders, Blackburn & Boucher 2013). Furthermore, identifying potential risk factors for injuries reduces the risk of injuries and medical bills as well. There is a lack of strength and conditioning research into the demands and the specific areas of development required to optimise performance and reduce the risk of common injuries in female netball athletes (Thomas et al. 2017).

The aim of our study was thus to identify risk factors for injury in elite netball players. More specifically, we investigated the question whether a range of pre-season assessments of morphological and skill-related components could predict injuries sustained in-season.

## Methods

A cohort study was conducted in which all participants underwent a fitness test battery pre-season and completed an injury report questionnaire when injured over the course of two seasons. A total of 110 players were eligible to participate over the 2017–2018 season. The U/18A high school league comprises five teams of 10 players each (*n* = 50). The Free State (FS) U/19 and U/21 teams had a squad of 36 players and the senior FS team had 24 players, respectively. Participants were recruited based on the inclusion and exclusion criteria presented in Table 1.

**TABLE 1:** Inclusion and exclusion criteria.

Inclusion criteria	Exclusion criteria
The player is a member of either the U18A high school FS netball league, FS U19, FS U21 or FS senior netball team and has played at least one match or attended training for the 2017–2018 season.	A player who has sustained an injury within 6 weeks prior to the study.
The player must be free from injury.	A player who sustains an injury during the season that is not netball-related or that occurs off the court.
The player must have given written consent or assent prior to the study.	Players who are unwilling or unable to give informed written consent or assent prior to the study.

FS, Free State; U18, under 18.

### Survey and laboratory testing

All participants completed a survey and underwent a battery of pre-season performance and functional tests. Injury data were obtained using a self-administered injury report questionnaire. The different teams were contacted on a weekly basis during the 2017–2018 netball seasons to ascertain whether any injuries had been documented.

All fitness and functional tests were screened for reliability and validity prior to construction of the test battery (Aragón-Vargas 2009; Bradley et al. 2014; Durandt et al. 2007; Gulgin & Hoogenboom 2014; Hotter et al. 2014; ISAK 2001; Ostojić, Stojanović & Ahmetović 2010; Plisky et al. 2006; Reiman & Manske 2009; Vanweerd 2013). Furthermore, a valid order of tests was gathered to determine an appropriate order of tests and the necessary recovery periods between tests (Attenborough et al. 2017; Harman & Garhammer 2008; McGuigan 2016). The fitness test battery was conducted over two consecutive days, consisting of a 3-min warm-up and static stretch routine with an emphasis on the lower body before starting with the testing each day. The fitness components that were completed included explosive power, strength and endurance, speed, anaerobic and aerobic tests.

Injury data were obtained using a self-administered injury report questionnaire based on a document drafted by the Rugby Injury Consensus Group to monitor the epidemiology of rugby injuries (Fuller et al. 2006, 2007; Pluim et al. 2009). Langeveld et al. (2012) adapted the questionnaire to specifically address netball injuries. The questionnaire was used to collect data on injury rates, timing (during match, pre-season, practice), type (acute/trauma, overuse, re-injury) and reason (contact with another player or not).

### Morphological measures

Body weight and height were measured using a calibrated electronic scale and a stadiometer. Players were required to empty their bladders before measuring their mass. They wore shorts and a T-shirt while being barefoot and stood against the height stand with their feet together, arms at their sides, buttocks and upper back against the wall and head facing straight in front. Body fat percentage was determined by using the sum of six skinfolds formula according to the International Society for the Advancement of Kinanthropometry (ISAK 2001) method of testing.

### Skill-related fitness

On day one, all players first completed their non-fatiguing tests, namely body weight and height and anthropometric measurements (skinfolds, girth and breadth measurements) before completing a 3-min warm-up and static stretches routine. Thereafter, the following tests were completed:

1. Active straight leg tests the participant’s hamstring and lower back flexibility in a dynamic movement pattern. The participant lies supine on her back with both legs extended. The examiner instructs the participant to actively raise her one leg straight into the air as high as possible. The dell rod is then placed vertically in line with the knee of the leg on the floor. According to the height that was achieved, a grading of functionality (0–3) is recorded (Vanweerd 2013).
2. The Star Execution Balance Test (SEBT) is a dynamic balance test that is functional for netball and is used to determine balance and postural control (Reid et al. 2015). The participant stands with hands on her hips on a ‘star sign’ in the centre. The participant is instructed to reach out with one foot while balancing on the other leg. Each participant must reach in eight different directions at 45° angles from the centre of the ‘star sign’. The participant is instructed to reach the furthest possible point with the toes, while maintaining her balance and returning to the start position. The distance reached is recorded on the measuring tape that is attached to the star sign. The test is conducted on both legs and measurements are compared (Reiman & Manske 2009).
3. The standing broad jump test is used to assess the participants’ explosive horizontal leg power. The participant stands behind a marked line with her feet shoulder-width apart on the ground. The participant must, without moving her feet, and only swinging her arms and bending her knees perform a two-foot forward jump and land with both feet on the ground simultaneously. The best of three trials maximum distance is recorded (Hotter et al. 2014).
4. Double- and single-leg vertical squat jump tests are used to assess vertical explosive power. The participant is instructed to stand next to the Vertec machine and reach up and touch the highest vane possible with one hand while keeping both feet firmly on the ground. This was recorded as the participant’s standing reach height. Participants perform the best of three trials double and single leg vertical jumps with sufficient rest between the trials. The maximal vertical jump is determined as the difference between the maximum height jumped and standing reach height (Hotter et al. 2014). The reliability and interclass coefficient (ICC) of the double leg vertical jump is excellent, (0.94) and coefficient variable (CV) 3.3 cm. Single leg has also excellent reliability of ICC of 0.96 and 0.91 for right and left leg, respectively, with CV 4.2 cm (Gonzalo-Skok et al. 2015).
5. The forward-jump test involves a unilateral jump in a forward direction followed by a step. Participants are required to step forward onto the take-off line and immediately perform a forward jump landing on the opposite limb to the take-off leg. During the jump, participants are required to touch a vane of the Vertec that is placed at a position equal to 30% of the subject’s vertical jump with the same arm as the landing leg. The Vertec is positioned at 50% of the horizontal jump distance and on the same side of the body as the arm that is required to touch it. Each participant is instructed to ‘stick’ on landing and remain still for 5 s. This test is netball specific. Lavipour (2009) found that more emphasis should be placed on horizontal explosive power (42% of all jumps) compared with vertical jumps (32%) in elite netball matches.
6. The jump with a turn in the air requires a unilateral jump in a forward direction with landing at 90° to the take-off direction, thus requiring the participant to control medio-lateral momentum. Participants are required to step forward onto the take-offline and immediately perform a forward jump, landing on the opposite limb. During the jump, participants are required to touch a vane of the Vertec with the opposite arm than the take-off leg. The participant is instructed to stabilise on landing and remain still in a self-selected position for 5 s. According to Lavipour (2009), it was decided to include a turn in the air and have the subject land laterally, because a movement frequency analysis found that lateral jumps (26%) and jumps with turns in the air (40%) are an important part of netball game play. Aragón-Vargas (2009) compared the methodology, reliability, validity and accuracy of four vertical jump tests and found the reliability to be > 0.97 and validity > 0.95.
7. The yo-yo test evaluates aerobic fitness over a period of time. Cones are used to mark out three lines 5 m (recovery), 0 m (start line) and 20 m (turn line). The participant starts on or behind the start line (0 m) and begins running 20 m when instructed by the voice on the compact disc (CD). The participant turns and returns to the starting point and must then complete the active recovery period of 5 m in (5 s) respectively, during which the participant must walk or jog around the other cone (5 m) and return to the starting point. A warning is given to the participant when she does not complete a successful to-and-fro shuttle and recovery shuttle in the allocated time. The participant is then told to stop the test. A participant’s final yo-yo score is the last successful shuttle she completed (Hotter et al. 2014).

The following tests were performed on the second day after again completing the 3-min warm-up and static stretches routine:

1. Horizontal pull-up was used to access pulling strength and endurance. Place a weight lifting bar in a squat or power rack. The participant’s arms must be fully extended and her body just off the ground. The participant grips the bar slightly wider than shoulder-width using an overhand grip. Her feet are flat on the floor and knees are bent at a 90° angle. The participant then lifts her hips so that her body is straight, and her arms are fully extended, so that she is hanging from the bar in a table-top position. The participant then pulls her body towards the bar until her chest touches the bar (nipple line aligned with bar) and lowers herself back down until her arms are fully extended. She continues doing as many repetitions as possible until muscle failure. The examiner records the total amount of correct repetitions completed (Hotter et al. 2014).
2. The press up assesses the participant’s upper body pushing and endurance strength. The participant starts in a plank position on her hands and knees off the ground. The examiner then places a closed fist in line with the participant’s chest. The participant then lowers her body to be in line with the fist and pushes up again until her arms are fully extended. The total amount of correct repetitions is calculated (Hotter et al. 2014).
3. Prone bridge assesses the participant’s core strength and endurance. The participant starts in a plank position with her elbows on the ground, feet approximately hip width apart, knees off the ground and body straight with no arching of the back or buttocks in the air. The head and neck should be facing towards the ground keeping the whole body aligned. The objective for the participant is to maintain a plank position for as long as possible whilst maintaining correct alignment. Once the participant can no longer keep her body straight, that is, she excessively arches or curls her back, then she stops the test. The total duration that the participant can maintain a good plank position is recorded (Hotter et al. 2014).
4. Sprints over 5 m, 10 m and 40 m are performed to test acceleration and speed. The aim of this test is to determine the acceleration and quickness of each participant. Electronic timing gates are placed at the start line 0 m, 5 m, 10 m and 40 m, to ensure that there is sufficient open space past the 40 m marker so that the participant can slowly decelerate and stop. The participant must start from a stationary position with a foot behind the line, no rocking or swaying movements prior to the start. The participant must conduct an adequate warm-up (5 min light jog, 10 min dynamic stretches) prior to the start of this test to prevent injuries. The participant completes the best of three trials with approximately 2 min – 3 min rest between attempts (Hotter et al. 2014).
5. The octo-repeater anaerobic fitness test assesses the player’s ability to perform repeated maximal sprints that incorporate a change of direction. This test mimics the game of netball by incorporating 10 m and 20 m repeated shuttle sprints that represent the maximum distance that the various player positions can run in the game. The octo-repeater test consists of 8 sprints starting at 25 s intervals. The participant completes 4 sets of 2 m × 20 m and 4 sets of 2 m × 10 m sprints, alternating between the 2 m × 20 m and 4 m × 10 m sprints, four times through, with 25 s rest intervals between each 2 m × 20 m and 4 m × 10 m sets. The participant must first complete the 2 m × 20 m sprint and 4 m × 10 m sprint described previously before completing the octo-repeater test. This is important in determining her fatigue rating over the test (Hotter et al. 2014).

### Injury report survey

On the day of fitness testing, a self-administered injury report questionnaire was completed by each player regarding the incidence, mechanism and severity of any injuries sustained during the previous season. In addition, throughout the study season, players sustaining an injury completed the injury report questionnaire. The first author explained the questionnaire to the players prior to completion.

### Data analysis

The primary objective of our study was to investigate whether variables measured in the pre-season (player morphology and skill-related fitness tests) could predict injuries during the in-season. The binary dependent variable (player had at least one injury vs. player had no injury) was analysed using both simple (univariate) and multiple logistic regressions.

In the univariate analysis, the potential predictors of injury were fitted one at a time as independent variables. For each model, that is, for each potential predictor of injury, a point estimate (PE) and associated 95% profile likelihood CI (95% CI) for the OR of injury, the corresponding likelihood ratio chi-square test statistic and *p*-value, the *p*-value of the Hosmer–Lemeshow goodness-of-fit chi-square statistic and the Nagelkerke *R*-square are reported.

Furthermore, a multiple logistic regression analysis was conducted where the binary dependent variable was analysed fitting jointly all those potential predictors of injury that were significant at least at the 10% significance level in the simple logistic regression analysis; in addition, the factor playing position was fitted in the multiple logistic regression model. (Playing position was fitted in the multiple logistic regression analysis because of the potential effect of that factor on injury risk and because the factor is associated with player morphology and is therefore a possible confounder). Again, PEs and 95% CIs for ORs were reported for the independent variables in the model, together with the associated *p*-values. In addition, the omnibus likelihood ratio chi-square statistic for the model, the *p*-value of the Hosmer–Lemeshow goodness-of-fit chi-square statistic and the Nagelkerke *R*-square were reported.

The above multiple logistic regression was followed by multiple logistic regression with stepwise model selection, in order to identify a set of significant predictors of injuries in the context of a multiple logistic regression model. The stepwise selection process started with the ‘full’ model (fitting all potential predictors of injury that were significant at least at the 10% significance level in the simple logistic regression analysis). At each step, the predictors associated with the largest *p*-value was removed from the model, providing that *p*-value was at least 0.1. Similarly, any predictors already removed from the model could be re-entered if the associated *p*-value was smaller than 0.1. The process terminated when no predictor was removed from the current model and none of the already removed predictors was entered into the current model.

The statistical analysis was carried out using the procedure LOGISTIC of the SAS/STAT (version 14.3) statistical software package (SAS 2017).

### Ethical considerations

Before the study commenced and participants were recruited, the study was approved by the Health Sciences Research Ethics Committee (HSREC) of the University of the Free State, South Africa (Ethics no. UFS-HSD 2017/0014). An informed consent form, and where necessary an assent form, was completed by all the participants participating in the study. The study included all U18 secondary school A league netball teams, FS U19 players, FS U21 players and senior FS netball players who participated in the 2017 and 2018 netball season for the respective teams.

## Results

### Anthropometric characteristics of study sample

A total of 77 participants, representing 78.6% of the possible 98 players, completed the skill-related fitness tests. The mean height was 170.9 cm (SD = 8.1, range 152 cm – 190 cm), and their mean body mass was 69.5 kg (SD = 11, range 47.1 kg – 108.2 kg). Furthermore, the participants’ body mass index (BMI) mean was 23.7 kg/m2 (SD = 3, range 17.9 kg/m2 – 36.2 kg/m2) and the mean fat percentage of the participants was 27.8 (SD = 4.8, range 19.3% – 39.7%). Detailed descriptive statistics of all fitness measurements are given in Sinclair, Coetzee and Schall (2020a). Eleven of the participants were goal shooters (GS), four GAs, 15 WAs, 15 Cs, 14 wing defenders (WD), seven goal defenders (GD) and 11 GK.

### Injuries

A total of 48 injuries was reported over the 2017 and 2018 season. Detailed data on these injuries were reported by Sinclair et al. (2020b). Table 2 presents, by age group and overall, the number and percentage of players who had at least one injury amongst the 77 players who completed the skill-related fitness tests.

**TABLE 2:** Players with fitness data and at least one injury in the 2017–2018 seasons, by age group.

Variables	Age group									
	Senior		U21		U19		U18A		All	
	*n*	%	*n*	%	*n*	%	*n*	%	*n*	%
Players with at least one injury	6	54.6	4	25.0	1	20.0	22	48.9	33	42.9
Total number of players in age group	11	-	16	-	5	-	45	-	77	-

### Simple logistic regression analysis

The potential association of anthropometric measurements and of skill-related fitness tests with the risk of injury is summarised in Tables 3 and 4, respectively.

**TABLE 3:** Morphological variables and player position as potential predictors of injury (univariate logistic regression).

Predictor (independent variable)	Likelihood ratio chi-square (*df* = 1)	*p*	Odds ratio	95% CI	*p* (Hosmer–Lemeshow)	Nagelkerke *R*-square
Mass (kg)	7.1166	0.0076	0.939	0.888–0.984	0.0633	0.1185
Height (cm)	2.9722	0.0847	0.951	0.894–1.007	0.8266	0.0508
BMI (kg/m^2^)	4.1842	0.0408	0.839	0.690–0.993	0.9211	0.0710
Body fat (%)	8.6494	0.0033	0.857	0.759–0.952	0.9181	0.1427
Player position (GK/GS versus other positions)	2.9545	0.0756	0.389	0.124–1.099	-	0.0539

*df*, degrees of freedom; CI, confidence interval; GK, goal-keeper; GS, goal shooter.

**TABLE 4:** Skill-related fitness as potential predictors of injury (univariate logistic regression).

Predictor (independent variable)	Likelihood ratio chi-square (*df* = 1)	*p*	Odds ratio	95% CI	*p* (Hosmer–Lemeshow)	Nagelkerke *R*-square
**Explosive power**						
Long jump (m)	0.0016	0.9677	1.044	0.131–8.521	0.2313	0.0000
Double-leg vertical jump (cm)	0.2624	0.6085	1.019	0.947–1.099	0.4744	0.0046
Single-leg vertical jump (cm)	0.4160	0.5189	0.974	0.898–1.055	0.6134	0.0072
Forward step and jump (cm)	0.2219	0.6376	1.013	0.959–1.071	0.8046	0.0039
Jump and turn (cm)	0.0366	0.8482	0.995	0.946–1.046	0.2301	0.0006
**Strength and endurance**						
Press-up (number)	0.2371	0.6263	0.985	0.926–1.045	0.2726	0.0041
Prone bridge (s)	0.6350	0.4255	1.004	0.995–1.013	0.7179	0.0110
Horizontal pull-up (number)	0.0071	0.9329	1.004	0.923–1.091	0.8582	0.0001
**Speed tests (m)**						
5 sprint (s)	1.3766	0.2407	0.070	<0.001–5.678	0.9102	0.0238
10 sprint (s)	0.3946	0.5299	0.333	0.009–10.046	0.8376	0.0069
40 sprint (s)	1.8160	0.1778	0.501	0.156–1.343	0.7193	0.0313
**Anaerobic (octorepeater test)**						
Total time (s)	1.5121	0.2188	0.954	0.875–1.027	0.3190	0.0261
Perfect time (s)	1.4757	0.2244	0.949	0.863–1.031	0.1424	0.0255
Fatigue index (%)	0.1016	0.7499	0.982	0.868–1.100	0.1651	0.0018
**Aerobic**						
Yo-Yo test (level)	0.8744	0.3497	1.232	0.799–1.962	0.6631	0.0152

*df*, degrees of freedom; CI, confidence interval.

### Anthropometric measurements

In the simple logistic regression analyses, the variables mass, BMI and fat percentage were found to be significant predictors of injury at the 5% significance level, whilst height and player position were significant at the 10% significance level. In all cases, the Hosmer–Lemeshow test did not show significant lack-of-fit, but the Nagelkerke *R*-square was small.

### Skill-related fitness tests

No skill-related fitness test was a significant predictor of injury. In fact, all *p*-values were greater than 0.2. Again, in all cases, the Hosmer–Lemeshow test did not show significant lack-of-fit, but the Nagelkerke *R*-square was negligible.

### Multiple logistic regression analysis

Only the morphological variables mass, height, BMI and fat percentage were identified as significant predictors of injury in the simple regression analysis (at a 10% significance level). Therefore, these variables and the factor playing position were fitted in the multiple logistic regression analysis, of which the relevant results are presented in Table 5.

**TABLE 5:** Morphological variables as potential predictors of injury (multivariate logistic regression, full model).

Predictor (independent variable)	Wald chi-square	*df*	*p*	Odds ratio	95% CI	*p* (Hosmer–Lemeshow)	Nagelkerke *R*-square
Global test	19.2754	10	0.0369	-	-	0.6093	0.2973
Mass (kg)	0.6909	1	0.4059	1.423	0.612–3.325	-	-
Height (cm)	0.8846	1	0.3469	0.728	0.369–1.414	-	-
BMI (kg/m^2^)	0.7447	1	0.3882	0.348	0.029–3.842	-	-
Body fat (%)	3.8155	1	0.0508	0.858	0.725–0.992	-	-
**Playing position**	6.8615	6	0.3338	-	-	-	-
WD versus C	-	-	-	2.358	0.455–13.144	-	-
WD versus GA	-	-	-	2.450	0.200–32.286	-	-
WD versus GD	-	-	-	0.175	0.007–1.667	-	-
WD versus GK	-	-	-	0.395	0.039–3.116	-	-
WD versus GS	-	-	-	1.682	0.195–15.377	-	-
WD versus WA	-	-	-	1.300	0.243–6.909	-	-

*df*, degrees of freedom; CI, confidence interval; BMI, body mass index; WD, wing defence; C, Centre; GA, goal attack; WA, wing attack; GK, goal-keeper; GD, goal defence; GS, goal shooter.

When fitting the ‘full’ model (all morphological variables and playing position jointly), only fat percentage was significant (*p* = 0.0508) at the 10% significance level. The global test for the ‘full’ model was significant (*p* = 0.0369), with a moderate Nagelkerke *R*-square and no significant lack-of-fit detected by the Hosmer–Lemeshow test.

When stepwise model selection was applied to this set of potential predictors, only fat percentage was selected in the final model (therefore, the OR 95% CI for the OR and *p*-value for fat percentage in the final model were the same as those in Table 3). Thus, amongst the predictors for injury investigated in our study, fat percentage was the strongest independent predictor, and fat percentage remained significant at the 10% significance level even when adjusted for mass and BMI in a multiple logistic regression analysis.

## Discussion

The univariate logistic regression analysis showed that both mass, BMI and fat percentage were significantly associated with injury, with the injury risk decreasing with increasing weight (or increasing BMI and fat percentage). Body mass index was significant (*p* = 0.0408), as was player position group (GK/GS vs. the other positions; *p* = 0.0756). The OR estimates indicated that with a 1 kg increase in weight, the odds of injury decreased by approximately 6% (OR of 0.94). Similarly, with a 1% increase in fat percentage, the odds of injury decreased by approximately 14% (OR of 0.86). According to Soh et al. (2008), the netball players with the lowest injury risk are the heaviest, tallest and those with the highest fat percentage. On the contrary, the less endomorph a player is, the less the chance of sustaining an injury (Ferreira & Spamer 2010). In our study, however, somatotyping was not performed.

Using a multiple logistic regression, only fat percentage emerged as a significant predictor of injury. Specifically, fat percentage remained a significant predictor, even when adjusted for mass (and BMI) in a multiple logistic regression analysis. Mass, on the other hand, whilst a significant predictor in the univariate analysis, did not remain significant when adjusted for the other morphological variables in the multiple logistic regression analysis.

Sinclair et al. (2020b) found that overall, half of all injuries were contact injuries. This fact could explain, at least in part, the protective effect of higher weight with regard to injury risk, as high weight might protect from injury and cause injuries in lighter players during contact.

No significant skill-related predictors of injury were found. Thomas et al. (2017) found that an increase in bilateral and unilateral strength and power will increase sprint and jump performance whilst improving lower limb control to reduce the risk of common injuries such as ankle and knee injuries. In addition, netball players should employ ‘HIT’ (high intensity training) modalities to improve their aerobic and anaerobic endurance to suit the physiological demands specific to their positions and competition demands (Thomas et al. 2017). According to Gamble (2011a), the body absorbs 6.8 times its body weight in ground reaction forces on impact of landing when receiving or intercepting a ball. This finding has implications for the need for development of muscle strength, eccentric strength, eccentric-speed strength capabilities for the players to overcome the absorption of landing forces, which directly impacts the risk of injuries. Furthermore, Gamble (2011b) suggest targeted intervention programmes to specifically address the common types of injuries in netball, namely ankle, knee, lower back and shoulder, must be completed in order to decrease these types of injuries from occurring. Decreased explosive power and increased biomechanical deviations could increase the likelihood of injuries throughout a netball season (Ferreira & Spamer 2010). Furthermore, McManus et al. (2006) found that players who trained for at least 4 h a week had fewer injuries than those who did not.

## Conclusion

Higher fat percentage, weight and BMI were found to be associated with lower risk of injury in our study. No significant skill-related predictors of injury were identified. The protective effect of higher weight regarding injury risk might be explained by the high proportion of contact injuries. If that is the case, then conditioning programmes that improve neuromuscular control (‘sticking’ the landing) and dynamic balance during explosive movements could decrease the risk of injury (Hopper et al. 2017; Humphrys et al. 2018).
